# A Scoping Review of Current Telehealth Interventions for Care Partners of Persons With Dementia

**DOI:** 10.1093/geroni/igaa057.621

**Published:** 2020-12-16

**Authors:** Leslie Tran, Robin Tarter, Allison Lindauer

**Affiliations:** 1 Oregon Health and Science University, Portland, Oregon, United States; 2 Oregon Health & Sciences University, Portland, Oregon, United States

## Abstract

Many burdens come with caring for someone with dementia, but telehealth interventions can provide information and support and reduce barriers to access. In this review we describe current telehealth interventions for family care partners of people diagnosed with Alzheimer’s disease or related dementias (ADRDs). We conducted a systematic literature search using PubMed. Inclusion criteria were peer-reviewed, data-based research articles focusing on technological clinical interventions, published within the past two years, with participants providing unpaid care to persons with a diagnosis of ADRDs. We identified 53 relevant articles, of which 13 met our criteria. Included studies fell into three categories: peer support groups facilitated by gerontological social workers and psychologists, psychoeducation and behavior modification interventions delivered by nursing professionals, and symptom management advising overseen by physicians. Different technologies were used including computers, iPads, smartphones, and smartwatches. The duration of interventions also ranged from four weeks to three months, representing varying approached to participant engagement. The majority of caregivers were women, non-Hispanic white, spouses. Based on our findings of a lack of diversity in the samples of extant studies, and the need for interventions tailored to specific stages of ADRDs, future researchers can design studies to address these gaps. Overall, the interventions’ effectiveness and participant satisfaction were high, resulting in improvements in burden, competence, and coping skills. Although every approach has its own strengths and weaknesses, we believe that the continued expansion of telehealth interventions will not only offer many benefits, but also transform the delivery of health care.

